# Reasoning and Knowledge Acquisition Framework for 5G Network Analytics

**DOI:** 10.3390/s17102405

**Published:** 2017-10-21

**Authors:** Marco Antonio Sotelo Monge, Jorge Maestre Vidal, Luis Javier García Villalba

**Affiliations:** Group of Analysis, Security and Systems (GASS), Department of Software Engineering and Artificial Intelligence (DISIA), Faculty of Computer Science and Engineering, Office 431, Universidad Complutense de Madrid (UCM), Calle Profesor José García Santesmases 9, Ciudad Universitaria, 28040 Madrid, Spain; masotelo@ucm.es (M.A.S.M.); jmaestre@ucm.es (J.M.V.)

**Keywords:** 5G, analysis, knowledge acquisition, pattern recognition, prediction

## Abstract

Autonomic self-management is a key challenge for next-generation networks. This paper proposes an automated analysis framework to infer knowledge in 5G networks with the aim to understand the network status and to predict potential situations that might disrupt the network operability. The framework is based on the Endsley situational awareness model, and integrates automated capabilities for metrics discovery, pattern recognition, prediction techniques and rule-based reasoning to infer anomalous situations in the current operational context. Those situations should then be mitigated, either proactive or reactively, by a more complex decision-making process. The framework is driven by a use case methodology, where the network administrator is able to customize the knowledge inference rules and operational parameters. The proposal has also been instantiated to prove its adaptability to a real use case. To this end, a reference network traffic dataset was used to identify suspicious patterns and to predict the behavior of the monitored data volume. The preliminary results suggest a good level of accuracy on the inference of anomalous traffic volumes based on a simple configuration.

## 1. Introduction

The rapid growth of network devices connected to mobile network infrastructures and the increasing demand of online Internet services have pushed new challenges for telecommunication operators. Future generation networks should guarantee several outstanding attributes [1] such as faster recovery times, higher traffic demands, higher levels of network Quality of Service (QoS), Quality of Experience (QoE), operational (OPEX) and capital (CAPEX) cost efficiency, among others. This generational change is driven by economic, societal and operational trends to conform the global network of the 21st century [2], being its first outcomes foreseen to be available by the year 2020. The main goal of next generation 5G networks is the efficient provision of services ensuring the agreed Service Level Agreements (SLAs) [3]. 5G networks should provide a sustainable and scalable network infrastructure to meet the exponentially-increasing demands on mobile broadband access [4], while leveraging competitiveness, standardization and faster innovation. 5G networks were designed to meet prominent requirements [5] in system performance, enhanced services provision, deployment times, as well as operational, energy and management efficiency. It leads to the definition of ambitious Key Performance Indicators (KPIs) such as higher peak data rates (10 Gbps), very low latency (5 ms), high mobility (500 km/h), a reduction in service creation time from and average 90 h to 90 min, among other disruptive capabilities [6,7]. Transversal 5G capacities are also security robustness and ubiquitous 5G access including in low dense areas, higher reliability and improvements to facilitate dense deployments. To fulfill the proposed requirements and objectives, 5G makes a suitable integration of supportive technologies [1,8], leading to the emergence of a novel mobile platform relying on new management paradigms and approaches as Software Defined Networking (SDN) [9], Network Function Virtualization (NFV) [10], Cloud Computing [11], Artificial Intelligence (IA), Self-Organized Networks (SON) [12], among others. Because of their design principles, the combination of these technologies will allow the development of a robust platform, flexible to be accommodated to different management schemas and more agile business models in more open environments with enhanced capabilities for virtualization, energy and spectrum efficiency, and service provisioning. In particular, the combination of SDN and NFV have opened a new network paradigm where several Virtualized Network Functions (VNF) can be deployed automatically from the network controller, levering to a fully network software-based management scheme [1,13]. Operational efficiency in 5G is strongly dependent on autonomic and self-management functions [14] provided at both control and data plane levels, where advanced real-time reactive and proactive network analysis procedures should be incorporated. In compliance with autonomic and cognitive networking architectures [15], network monitored data acquired by sensors in a 5G network enables the possibility to transform low-level metrics into context-aware analytics due to the insertion of machine learning and knowledge-driven approaches supported by software-based platforms [16]. Current research towards their integration is grounded in the Knowledge-Based Systems (KBS) and their adaptation to heterogeneous monitoring environments [17]. Regardless of the knowledge representation they implement, the proper functioning of their Inference Engines (IE) demands high-quality knowledge about the use cases domains, which is not easy to obtain at the emergent scenarios. This is due to different reasons, among others the presence of redundant information, difficulties when selecting the most significant metrics/facts, lack of validation, inconsistencies between sources, computational limitations or data protection policies. Because of this most of the solutions to these problems aims on specific use cases, hence reducing their scope and ignoring the rest of the particularities in the 5G environments [18,19,20].

With the aim to contribute to a smarter 5G network management, this paper proposes the Reasoning and Knowledge Acquisition Framework Analytics designed with a machine learning approach. It is composed by functional elements with data discovery features, prediction capabilities, pattern recognition methods and knowledge inference techniques to acquire and generate knowledge about the underlying mobile network with the purpose of analyzing and inferring potential events or situations that might affect the desired operational levels of a 5G network. The introduced software-based proposal is aligned with the principles and supportive technologies of 5G, and brings analytical capabilities to deploy a self-organized autonomic network. The research has been conducted under the SELFNET Horizon 2020 project [21], bearing in mind its scope and objectives.

The major contributions of this paper to the sensor and multi-platform information processing, and the advances on knowledge-based management approaches upon monitored 5G infrastructures are summarized below:

- *In depth study of recent 5G-related research contributions*. Several research topics related with the definition of the 5G technology, European research projects and network incidence management approaches have been studied with the purpose of laying the foundations, design principles and architectural components of the introduced framework. On the one hand, 5G provides research challenges to be addressed by innovative network architectures that are still under development, many of them targeted by recent European research projects. In particular, the SELFNET project offers a baseline architecture for network self-management in 5G mobile networks, under which the proposed framework was developed. On the other hand, advances on network incidence management in dynamic scenarios have been considered. Special interest has been put on the Endsley inspired architectures, since they motivated the adoption of a situational-awareness model in the proposed framework.
- *A novel reasoning 5G-oriented architecture*. A novel framework composed by functional elements arranged on an orchestrated workflow is proposed to enable reasoning capabilities in a 5G network. As a result, the framework generates conclusions about the 5G network status. The introduced architecture distinguishes two types of knowledge: procedural and factual. Procedural knowledge corresponds to the use case configuration loaded to the system. Initial factual knowledge is acquired by discovery methods applied on data previously collected by several sensors distributed along the 5G network, whereas factual knowledge is generated by the prediction, pattern recognition and knowledge inference modules introduced in this proposal. Thereby, the framework approaches the perception, comprehension and projection steps of the Endsley model.
- *Instantiation of the framework*. An instance of the proposed framework has been created to enhance the understanding of the proposal. To this end, well-known multiplatform open source technologies and a battery of prediction and machine learning algorithms have been integrated in accordance with the framework design. In addition, publicly available datasets were applied to allow its experimental replicability. The generation of knowledge was successfully demonstrated in a datacenter-oriented use case, even though the current instance of the framework can be applied to several use cases just by modifying its configuration.
- *Comprehensive experimentation on a real use case*. To assess the accuracy of the instantiation, a set of experiments have been conducted. They were oriented either for the evaluation of the pattern recognition and prediction modules; and for the evaluation of a real use case. Prediction and pattern recognition features exposed good accuracy levels when applied over the reference datasets. Likewise, a particular use case configuration to generate conclusions about traffic behaviour has been tested. The experiments were conducted on real network traffic samples where the inference of suspicious network traffic volumes in a datacenter exposed good precision rates contrasted with the real reference scenario.

The paper is structured in six sections, this one being the introduction. Section 2 describes the background knowledge for this proposal. In Section 3, the Knowledge Acquisition Framework for 5G network Analytics, and an example of its instantiation is described in detail. Section 4 presents the experiments conducted to validate the implemented framework on a real use case. Section 5 summarizes and discusses the results obtained at the experimental stage. Finally, Section 6 remarks the conclusions and future work derived from the performed research.

## 2. Background

The following describes the key elements and the background to be kept in mind in order to properly understand the rest of the paper. Among them it is worth highlighting the recent progress toward the deployment of fifth generation networking technologies, the role of the SELFNET project in their development, and some of the most common approaches focused on the management of network incidents.

### 2.1. Research in Fifth Generation Networks

Research initiatives in 5G have been conducted to cope with the challenges of new generation mobile networks. These approaches are worldwide fostered and funded by different organizations, such as the European Horizon 2020 programme [22] (preceded by FP7), the IMT-2020 [23] group in China, 5G Americas [24], among others. In the European context, some FP7 projects such as METIS [25], T-NOVA [26], UNIFY [27] and COWD [28] have contributed to lay important research lines for other initiatives. For example, the project METIS (Mobile and wireless communications Enablers for the Twenty-twenty Information Society) proposed and agreed European foundations for the development and standardization of global mobile and wireless communication systems. Likewise, T-NOVA project takes advantage of SDN and NFV; and focuses on the automated deployment of Network Functions as a Service (NFaaS) on virtualized infrastructures. It is accomplished by the design and implementation of a platform enabled for the provisioning, configuration, monitoring and optimization of virtual resources. Complementary, a dynamic platform for automated end-to-end service delivery is explored by the UNIFY [27] project. It targets the dynamic and orchestrated provision of services in cloud environments with optimal placement of service components across a 5G infrastructure. Unlike T-NOVA and UNIFY, which rely on the potential of SDN, NFV and cloud technologies; the CROWD project is intended to significantly increase wireless mobile network density; guaranteeing QoE, resource optimization and energy consumption reduction. Promoted under the 5G-PPP Phase 1 projects, some outstanding H2020 initiatives, encompassing several research fields, are METIS II, COGNET, CHARISMA, 5G-ENSURE and SELFNET. They are, at the same time, devoted to set collaboration partnerships to enhance current and future outcomes. The METIS II [29] project aims to develop a seamless integration of 5G radio technologies by inserting a protocol stack architecture that addresses regulatory and standardization challenges, hence providing a 5G collaboration framework. A smart management of the Cloud Radio Access Network (C-RAN) is addressed by CHARISMA [30] project, leading to the smart deployment of network services through the intelligent management of C-RAN environments and the Radio Remote Head (RRH) platforms to target low latency, higher density, increased data rates and efficient spectral and energy management. Incidence management challenges in 5G are targeted by the 5G-ENSURE [31] project, covering a wide range of security and resilience concerns in 5G, aiming on standardization, privacy and architectural issues; with the purpose of providing reliable security services with “zero perceived” downtime. Network Functions Virtualization and service chaining are a disruptive capability in 5G where the SONATA [18] project addresses the challenges regarding their development and deployment by enabling a Software Development Kit (SDK) and an orchestration framework to offer a novel platform for the rapid deployment of services and applications. A machine learning approach to allow autonomic network management is proposed by the COGNET [19] project which introduces self-organizing capabilities based on network monitored information applied to machine learning algorithms in order to predict the demand and provision of network services dynamically adapted to the current network state as a result of the identified network errors, fault conditions, security and other related issues. Likewise, SELFNET [21] evolves the concept of self-organizing networks by developing a framework for autonomic network management. SELFNET is further explained in the next section. Several other research ongoing efforts in the 5G domain are described with more detail in [3,32], which share the common vision to fulfill the proposed 5G KPIs to enable a feasible worldwide adoption of this emergent technology.

### 2.2. SELFNET

**Figure 1:**
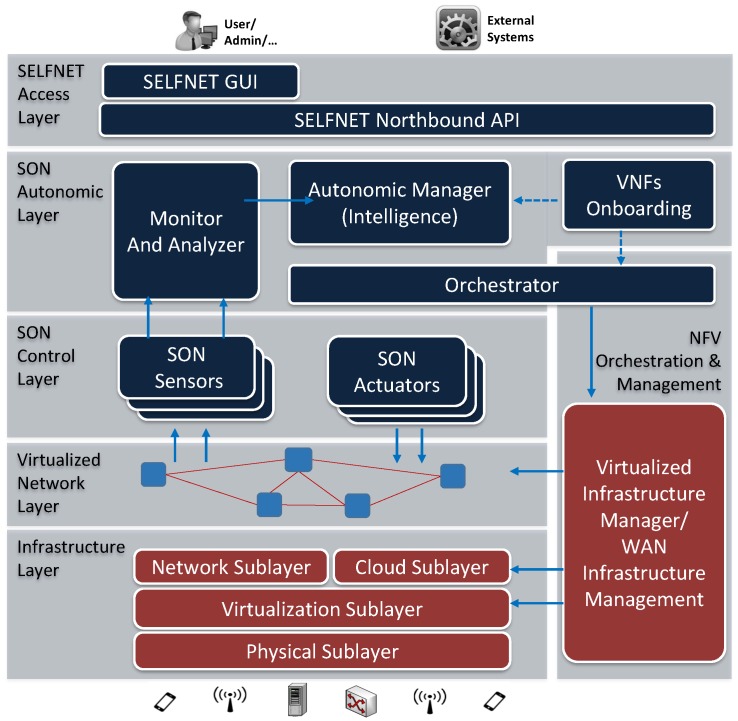
The SELFNET project architecture.

SELFNET (Self-Organized Network Management in Virtualized and Software Defined Networks) was proposed with the main goal to develop a smart autonomic network management framework to provide self-organization capabilities in mobile 5G networks. This project status is in an ongoing stage and it is funded by the Horizon 2020 programme. SELFNET reference architecture [33] relies on the principles of SDN and ETSI-NFV to allow an intelligent management of different network functions intended to detect and automatically mitigate a range of common network problems in 5G networks, such as network congestion, transmission delays, link failures, among others. SELFNET defines three major use cases: self-protection [33], self-optimization [34] and self-healing [35]. Each of them distinguishes several scenarios in which network analysis, decision-making and action enforcement are required. The architecture (Figure 1) is composed by functional layers. In the bottom part, the Infrastructure Layer holds the physical resources required to deploy and instantiate virtualization functions (computing, networking and storage). On top of that, the Virtualized Network Layer holds the instantiated network elements that compose individual network functions (NFs) as well as chained NFs deployed as network services on the virtual topology. In the next level, the SON Control Layer contains the different SON sensors for data collection, and the SON actuators for the enforcement of the detected countermeasures. The upper level is for the SON Autonomic Layer, which is the core of SELFNET intelligence, committed to monitor relevant data to analyze and acquire knowledge about network behavior, diagnose the cause of network potential failures and decide the best actions to be enforced to accomplish the system goals and the agreed service levels. A supportive architectural component is the NFV Orchestration and Management Layer, which allows an automated and efficient orchestration of NF deployment in the network infrastructure. In the highest level, the Access Layer allows the interaction of SELFNET with external users, network administrators or external systems through suitable Application Programming Interfaces (APIs) for an efficient management of the system.

SELFNET embraces the autonomic management paradigm by integrating enhanced monitoring features, prediction algorithms, pattern recognition strategies, machine learning capabilities, orchestration of virtual functions and service chaining to conduct a SON approach focused on the identification of the current network behavior, the decision of the best mitigation responses against the identified network problems, and the deployment of the corresponding actuators in the infrastructure. To facilitate the operational context comprehension based on the Endsley situational awareness model, a three-step schema of Monitoring, Aggregation and Correlation, and Analysis is proposed, which, maps the Monitor and Analysis component of the SON Autonomic Layer:

- Monitoring has the main objective of collecting a wide range of low level metrics and events from the physical and virtual network infrastructure, as well as from deployed sensors.
- Aggregation and Correlation methods reduce the amount of monitored information, by inferring aggregated metrics about a specific network domain. Meanwhile, events are correlated and filtered to avoid redundant or non-sensitive information.
- Analysis is aimed for reasoning and knowledge acquisition about the operational network context deducted from the analysis of aggregated monitored metrics. This process is accomplished by pattern recognition capabilities, prediction methods, and knowledge inference procedures in order to deduce conclusions regarding potential network failure or degradation scenarios projected from the observations.

Through the rest of the paper, the proposed reasoning and knowledge acquisition framework is aligned with the SELFNET Analyzer component. Its design and implementation is deeply covered in Section 3.

### 2.3. Network Incident Management

In the last four decades, a great variety of contributions related with incident management have been published. Some of them are classified and reviewed in depth in [36,37], where a marked trend toward the adoption of classical information security risk management schemes is emphasized. They pose different lines of research, raging from the mere definition of the risk management terminology [38], to the proposal of practical guidelines for their mitigation [39]. The first of these incidence management groups of publications lies in the foundation of conceptual security models, as is the case of the well-known CIA-Triad [40], the Parkerian Hexad [41] or the Cube of McCumber [42]. On the other hand, several authors focused on the study of how mitigate potential threats, hence establishing the basis for standards [43,44], guidelines [45] or platforms [46]. As indicated by Ben-Asher et al. [47] a greater specialization in this area of research and its applications representatively improves the effectiveness of defensive deployments, which is certainly a very important step toward bring self-organizing capabilities to 5G environments. However, it is important to highlight that in the networking context, an incident do not only reports a risk. In fact, they occur in connection with something else, which may be the result of a security threat, but also the outcome of the deployment of countermeasures, or even the variation of certain network management policies, such as enabling additional bandwidth, the discovery of new devices or the optimization of some resource usage, in this way also bringing feedback about the effectiveness of the self-management actions. Therefore understanding the nature of an incident and its impact usually requires a comprehensive overview of the state of the network and the different cause-effects monitored in them. Most of the recent proposals for network incident management combine the conventional risk management schemes with the situational awareness model proposed by Endsley [48]. According to Endsley, situational awareness means “to have knowledge of the current state of a system, understand its dynamics, and be able to predict changes in its development”. Because of this her model distinguishes three major steps: perception, comprehension and projection; where the first of them is related with monitoring the environment and the discovery of initial facts, comprehension aims on the inference of knowledge, hence generating new facts from the observations, and projection is related with the prediction of the environment status. Note that the conventional model introduces feedback between data processing stages, in this way allowing learning and improving decision-making. As discussed in [49], the Endsley model demonstrated perform effectively in complex and dynamic scenarios, where the diagnosis from incidents highly depends on their context. Throughout the bibliography it has been successfully combined with risk management models [50] and adapted to networking environments, consequently the research community coined the term NSSA (Network Security Situational Awareness) [51]. Its adaptation to 5G started with project like 5G-ENSURE [31] which were mainly inspired in the risk assessment and management approaches. More recently, SELFNET [21] adopted the situational awareness paradigm based in the research of Barona et al. [52], which described a framework for hybridize the Endsley model, information security risk management and self-organizing networking. The perception stage of SELFNET was described in depth in [53], and a first approach toward orchestrating the activities related with comprehension and projection was published in [54].

## 3. Reasoning and Knowledge Acquisition Framework

The introduced framework is aimed to grant analysis capabilities to a 5G network by the proper instantiation of its components at the management plane, decoupling data analysis logic from specific data extraction procedures carried on at the lowest architectural levels of the network. It allows the insertion of several prediction algorithms, pattern recognition capabilities and production rules to generate meaningful knowledge that will enhance decision-making processes from the performance and efficiency perspective. Thereby, the framework provides the advantage to express metrics gathered by sensors (initial facts) according to a knowledge representation language in order to deduce conclusions about possible network scenarios driven by the Endsley approach [52]. Perception, comprehension and projection steps are performed to understand the system state. The discovery of initial facts, which corresponds to previously monitored data, accomplishes the perception step. Reasoning involves both perception and comprehension, whereas prediction approaches the projection step. The deduced final facts (conclusions) are described in the form of symptoms related with each use case. Bearing this in mind, it is possible to assert that this framework provides a symptom-oriented situational awareness bounded by the configuration defined for each use case. With this purpose, the following subsections introduce the design principles and constraints assumed for knowledge acquisition, an in depth description of the proposed architecture, and a detailed explanation of the framework instantiation to enhance the comprehension of the proposal.

### 3.1. Design Principles and Constraints

The following design requirements and assumptions have been kept in mind at both design and implementation stages of the reasoning and knowledge acquisition framework.

- *Scalability*. The proposed framework must accommodate the 5G design principles, and in particular, those associated with scalability, such as “Extensibility by design”, “Expandability by design” or “Multi-level scalability by design” [33], through the combination of scalable modular design, open interfaces and APIs to enable third parties to create their own automatic network management services.
- *Support of use case onboarding*. The knowledge acquisition framework adopts a use case driven research methodology. Because of this it is required that from design, it must support the onboarding of new different use case specifications. Given the heavy reliance of the tasks performed with the characteristics of use cases, the basic definition of the observations to be studied (knowledge-base objects, rules, prediction metrics, etc.) must be provided as factual knowledge by use case operators, thus being the framework scalable to alternative contexts. In addition, use case operators must provide procedural knowledge, thus configuring the analytic tasks to be performed per use case. More information about these knowledge representations is detailed in [55].
- *Reference datasets*. Laskov et al. [56] realized two essential observations necessary to understand the different strategies for acquiring reference knowledge and to decide the most appropriate for each use case: firstly, it must be taken into account that labeled samples are very difficult to obtain, a situation that can be aggravated if the sensor operates in real time, and/or on monitoring environments where is not possible to extract all the data; on the other hand, there is no way of collecting labeled samples which cover every possible incidence, so the system is potentially vulnerable to unknown situations. To these difficulties it is added the problem that there are no collections of traffic captures in 5G networks, and that the existing datasets of current traffic traces often have drawbacks such as lack of completeness or labeling errors. Because of this, the proposed framework does not go deep into the issue of the innate knowledge acquisition. The current approach assumes that the reference datasets are provided by skilled operators or by accurate machine learning algorithms, which therefore are valid for the specified use cases.
- *Granularity*. 5G environments are complex monitoring scenarios where large amounts of sensors collect information about the state of the network in real time. In SELFNET all this information is processed in the Aggregation sub-layer, which provides the necessary metrics to infer knowledge from them. However, this information is not raw processed. As described in [54], it is compiled into Aggregated Data Bundles (ADBs), which summarize all the system information observed over a time period related with the previously declared uses cases. The length of the observation period defines the data granularity, which may be determinant for the proper functioning of certain uses cases. However, the decision of the best granularity is out of the scope of this paper.
- *Stationary monitoring environment*. By definition, the features monitored on a stationary scenario are similar to those considered when building data mining models. The assumption of operating on a stationary monitoring environment entails ignoring in terms of learning process any variation in the characteristics of the information to be studied, such as dimensionality or distribution. The main disadvantage of this approach is the loss of precision when such changes occur, in large part because the initially performed calibrations are not adapted to the current status of the network. On the other hand, their proper accommodation tends to retain the acquired calibration at the expense of addressing many other issues, emphasizing among them to discover relevant variations in the data nature, calibration upgrades based on the new features, or improvement of the original datasets [57]. Being aware that the last approach poses important challenges, and in order to facilitate the understanding of the proposed research, all those aspects related to the management of the non-stationary characteristics of the information are overlooked.
- *High dimensional data*. When the dimensions of the data to be studied are more extensive than usual, it is possible that some reasoning and knowledge acquisitions implementations lose effectiveness, either in terms of efficiency or accuracy. Because of this, the bibliography provides a wide variety of publications focused on the optimization of this kind of processes, as is discussed in [58]. Note that the battery of algorithms included in the current instantiation of the framework does not adapt any of these contributions, which does not mean that it is incompatible with them. However, throughout the document the risks of operating with high dimensional data are not taken into account, in this way postponing this problem to future instantiations.

### 3.2. Architecture

**Figure 2:**
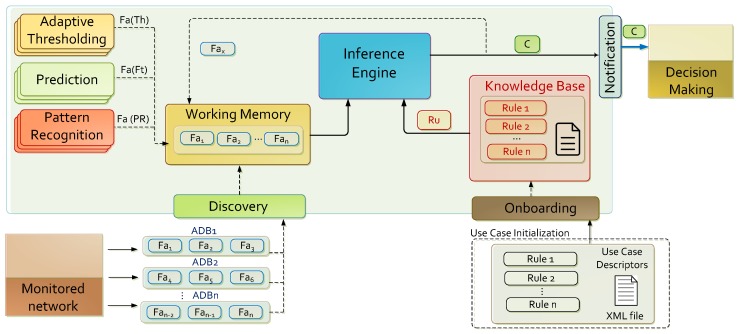
Knowledge acquisition framework for 5G environments.

The proposed framework is composed by the functional elements illustrated in Figure 2. They are settled down to interact as providers and consumers of facts not only discovered from monitored data, but also deduced by reasoning procedures. The architectural elements of the framework are pipelined sequentially as: Onboarding of use cases, Discovery, Pattern Recognition, Prediction, Adaptive Thresholding, Knowledge Inference and Notification. These elements are coordinated by the orchestration strategy defined in [54]. Hence, and assuming the design principles previously stated, the proposed framework brings analytic capabilities focused on acquiring knowledge from the network metrics (initial facts), and deduces conclusions (final facts) such as likelihood of the network being attacked, anomalous congestion levels, among others.

The Discovery component obtains information, represented as facts, gathered by network sensors in the lower levels of a 5G architecture by monitoring, data aggregation and correlation procedures. It exempts the framework to the need of dealing with network technology-dependent protocols or interfaces, and allows assuming that the constraints inherent in the monitoring environment (for example, ciphering, privacy protection politics, etc.) have no impact on the effectiveness of the proposal, since they have been previously managed at lower data processing stages. New knowledge is acquired by the Inference Engine based on the collected facts stored in the Working Memory, and permits the inference of conclusions about the network status by applying production rules configured in the Knowledge Base. Conclusions are expressed as symptoms, reflecting situations that might affect or compromise network operability or a degradation of the agreed service levels. The framework also facilitates a situational awareness projection of the network through the Prediction and Adaptive Thresholding components, by calculating predictive metrics and forecasting intervals that allow pro-action responses over the predicted scenarios. Likewise, the Pattern Recognition component implements some of the recent paradigms of Artificial Intelligence, among them machine learning, data mining, classification or novelty detection methods.

To deal with scalability, each analytic component is designed to be executed independently, exchanging only input and output data between them. From the instantiation perspective, it is accomplished through the implementation of buffering data structures or by the deployment of message broker tools (such as Apache Kafka [59] or RabbitMQ [60]). The underlying technology to instantiate each component (i.e., Weka, Drools, among others) should be also scalable by design at both vertical and horizontal levels in order to accommodate several deployment strategies when computing resources demands are variable. In this way, the proposed framework is aligned with the principles of 5G by developing a scalable solution adapted to the latest trends in the control and data planes of 5G mobile architectures [61], hence allowing its deployment at the management side. The role of each framework component is explained in detail in the following subsections.

#### 3.2.1. Initial Knowledge and Notification

The Knowledge Base is filled from data acquired from the use case definitions at use case Onboarding. Because of this, use case operators may declare procedural knowledge such as inference rules Ru, prediction actions Ft, etc. and specify factual knowledge such as Objects *O*, Facts Fa, Thresholds Th, etc. in compliance with the use case descriptors defined in [55]. They also provide the reference datasets required for machine learning actions. Factual knowledge is gathered by the Discovery component, which periodically receives ADBs which summarize the acquired observations. From the loaded metrics and events, the knowledge acquisition framework build facts (Fa). If they are required for prediction, pattern recognition or adaptive thresholding, these observations are inserted in the temporally stored time series. Note that independent facts are removed at the end of the ADB processing, as well as the new knowledge acquired from them. It is remarkable that the aforementioned procedural and factual knowledge represent the inputs of the proposed framework. However, it is also worth mentioning that new factual knowledge is internally generated by the Prediction, Pattern Recognition and Adaptive Thresholding components for inferring new knowledge. The set of actions in Notification reports the final facts (conclusions) deduced by the reasoning framework. This step packs the conclusions by inserting or modifying the related meta-knowledge to accommodate contextual information, such as facts location, timestamps, output format representation (i.e., JSON), among others. Once an ADB is fully analyzed, these actions also erase and restart the auxiliary functionalities on the analytics and several data structures. Only the information and buffers required for building time series with data to be extracted from future ADBs is temporally persistent.

#### 3.2.2. Prediction Module

This component drives the inference of knowledge related with prediction facts built from the monitored data. Its main purpose is to insert facts about forecasted metrics in the Working Memory, and the observation of variations of interest such as discordant values or relevant decreases or increases on the analyzed data. Although the framework does not support persistent storage, several data must be temporally preserved to allow registering time series and information needed for enhancing the decision and calibration of the prediction algorithms. Then the forecasting strategies must be cautiously selected and adjusted with the purpose of providing the more accurate results. Once the predictions are calculated, the system includes the discovered knowledge (facts) into the Working Memory. Note that this data processing stage depends on synchronous ADB loading where time series are fed with observations fetched from the ADBs. It requires two types of knowledge: procedural and factual. Procedural knowledge is provided via use case onboarding in their data descriptors. Likewise, factual knowledge is acquired by the Discovery component, when new ADBs are loaded. Once the time series with the required length are built, several prediction methods are evaluated to decide the most accurate algorithm fitted to the given time series. The selection of the best forecasting methods entails several steps. Once data is acquired, a time series of size *N*, and the forecasting horizon *T* are taken as input parameters for a preprocessing task. The last *T* elements are subtracted from the original time series and the remaining N−T elements are used for forecasting. In the meantime, the subtracted *T* elements are reserved to be used for evaluation. Parameter calibration takes place for all the forecasting algorithms included in the framework, each one has a variable number of parameters. Every individual parameter can be tested with different values, thus allowing a forecasting algorithm to be run with different calibration coefficients.

#### 3.2.3. Adaptive Thresholding Module

Throughout the tasks involved in the reasoning process, it is necessary to define under what circumstances an observation about the monitoring environment must conditionate the inference of new knowledge. This is a complicated challenge, which must take into account both the situational awareness of the monitoring scenario and the specific use cases on which the self-organizing deployment for incident management has been implemented. Therefore, the instantiated adaptive thresholding strategies must pose dynamic solutions, subject to the changes in the different features of the scenario on which they operate, and must be configurable according to the level of constraint that operators decide (note that the restrictiveness may also be stablished by machine learning approaches). Because of this, the calculated thresholds act on any source of knowledge of the Knowledge Inference engine (e.g., Discovery, Prediction, Pattern Recognition, etc.), or may be part of the production rules. This makes the results they provide considered as factual knowledge by the knowledge-based of the framework, being Adaptive Thresholding and additional knowledge acquisition step dependent of the rest of the components of the proposal.

#### 3.2.4. Pattern Recognition Module

The Pattern Recognition component of the proposed framework operate at two different stages: training and discovery. At training, the knowledge representation to be taken into account, as well as the description of the pattern recognition actions are included into the procedural knowledge according to the use case specifications. This step involves generating/loading reference datasets and construction of the best models in function of the most relevant data features on the sets of metrics to be analyzed. The training step may take place in two moments of the analytic process. Firstly, models from reference data can be built before operating on real monitored samples. On the other hand the training step may operate at runtime, so the reference samples are gathered from the first observations on the monitoring environment. At the discovery stage, the knowledge acquisition framework launches the pattern recognition actions defined by the use case operators. Samples are constructed from the aggregated metrics observed, and they are analyzed based on the models built at training stage. The framework at least allows two pattern recognition actions: classification and novelty detection. When classifying, a reference dataset is loaded before the monitoring of the protected environment. A battery of classification algorithms is executed in concurrency, which are properly calibrated and combined as an ensemble of models [62,63]. Then the most accurate classifier is identified by cross-validation on the reference sample collection [64], and it is applied at the discovery step. On the other hand, the novelty detection actions are usually defined as the tasks of recognizing that test data differ in some respect from the data that are available during training, which also can be generalized as one-class classification [65]. These methods are usually applied to solve problems where the analytic system was provided by a long and complete collection of reference samples (commonly “normal” observations), and it is required to decide if the observed data can be tagged as belonging to the population on the reference dataset, or if it has “discordant” nature. The proposed framework implements novelty detection similarly to classification, in this way also based on an ensemble of sensors. However, in this case, the training stage considers data observed at runtime from the monitoring environment. Particularly, the first monitored metrics define the reference dataset, and hence are tagged as normal observations. The length of this collection is previously defined by the use case operators. It can be manually delimited or decided by the results of the cross-validation scheme; in the second case, if the accuracy is greater than certain threshold, the model is considered acceptable and there is no need to process additional samples.

#### 3.2.5. Knowledge Inference Module

The knowledge inference component allows deducing information from previous observations (facts) based on procedural knowledge represented as rule sets. In order to align the decision strategy of which rules should be activated and when, with the previously assumed design principles and requirements, the implemented approach is driven by production rules. This facilitates the deployment of a modus ponens (forward chaining) decision scheme where attributes enable the deduction of goals, which are final facts encapsulated as symptoms before their report to the decision-making layer. It should be kept in mind that throughout the bibliography, Rete algorithms are the most popular and proved proposals to address the efficient implementation and execution of forward chaining on complex monitoring environments. Created by Forgy [66], these methods separate the rule management into two steps: rule compilation and runtime execution. The first stage describes how the rules in the Working Memory are processed to state an efficient discriminant network, where upper nodes tend to present many matches, in contrast with the lower elements (the bottom are termed terminal nodes). The main reason on building this structure is to optimize the number of triggered rules, while at runtime, the previously built network allow inferring the new knowledge. Thereby, Rete algorithms are appropriated to address the knowledge inference purposes of the proposed framework.

### 3.3. Instantiation

As an illustrative example of instantiation of the proposed framework, this subsection describes how it has been deployed with the aim on contributing with the management of a real network. Its contribution is focused on the recognition of discordant behaviors based on analyzing the variations of the traffic volume, which borne in mind the prediction of their evolution, the construction of adaptive thresholding to decide when they may be considered unexpected, and novelty detection based on several distances and similarity measures. It is important to emphasize that the instantiated solution could be replaced by an alternative implementation and still achieving similar results. Nevertheless, with this subsection it is intended to describe a simple, didactic and scalable solution, that provides a greater understanding of the proposed framework and draft several basic guidelines for its adaptation to other problems. With this purpose, the following introduces the implementation of each of the aforementioned reasoning and knowledge acquisition components considered at the experimentation.

#### 3.3.1. Initial Knowledge and Notifications Implementation

The initial knowledge of the instantiated framework is directly provided by the use case operators, hence postponing auto-calibration and other machine learning approaches for future work. It includes specific information about what activities must be monitored, what knowledge acquisition actions should be accomplished and what kind of reasoning must be carried out; the latter is guided by production rules and the inference of conclusions about the state of the network. Therefore, it can be said that the initial knowledge is the configuration of the framework and the strategy of acquiring the initial facts to be analyzed. These facts arrive to the system compiled as ADBs, which gather the information monitored in certain time periods. As stated before, the different sensors deployed on the 5G infrastructure are considered the most important information providers. For this framework instantiation the initial metric to be studied is the traffic volume per observation, which is assumed to be already reported by the sensors. With this collected data, the framework creates the required time series, enabling the possibility to apply prediction methods, i.e., to estimate the traffic volume at the coming observations according to a given forecasting horizon. The system is also configured to build adaptive thresholds based on the forecasts. They allow to identify if the observed traffic volume differs significantly from the predictions. Herein, those traffic observations are labeled as *unexpected*. On the other hand, the pattern recognition component is configured for novelty detection based in analyzing difference distances and similarity measures between each monitored observation and that of an immediately preceding monitoring period. Note that his action requires building one-class classification models, which demand a reference dataset. It is obtained from the first observations made, so directly loading an external dataset is not required. The discordant observations are in this stage labeled as *fluctuations*. On the other hand, the Knowledge Inference component is configured by production rules to conclude that an observation marked as *unexpected* and *fluctuation* is a suspicious event, hence being notified as a symptom. The findings are reported through a message broker software to be consumed by external sources, i.e., to perform more complex decision-making procedures. Table 1 summarizes the initial knowledge and notifications of the proposed framework instantiation considered at the experimentation.

#### 3.3.2. Prediction Implementation

The current instantiation of the proposed framework does not support objects with multiples values. Because of this, the adapted battery of forecasting algorithms only considers univariate time series. This does not mean that this capability cannot be included in future instantiations, but it has been considered that working with a simpler instantiation facilitates the comprehension of the prototype, as well as the specification of new use cases. Two families of well-known forecasting methods are implemented: moving averages and exponential smoothing, as detailed in Table 2. They process the time series of monitored metrics in concurrency, and the decision of the best algorithm grounds on considering the minimum Symmetric Mean Absolute Percentage Error (sMAPE) [67] as the forecasting error measure. sMape considers the set of real subtracted N−T values *X* and the *T* forecasted values as inputs. It is described by the following expression:

(1) $$sMAPE=200\%\sum_{t=1}^{n}\frac{|F_{t}-X_{t}|}{|F_{t}\left|+\right|X_{t}|}$$

where *X* represents the real time series values and *F* are the forecasted values estimated for the given observations.

#### 3.3.3. Adaptive Thresholding Implementation

To evaluate the prediction errors, two adaptive thresholds are constructed: an upper threshold $Ath_{up}$ and a lower threshold $Ath_{low}$. They establish the Prediction Interval (PI) of the observations, which is defined in the same way as is usually performed in the bibliography [76], hence assuming the following expressions:

(2) $$Ath_{up}=p_{0}+K\times \sqrt{var(E_{t})}$$

(3) $$Ath_{low}=p_{0}-K\times \sqrt{var(E_{t})}$$

where $E_{t}$ is the prediction error in *t* and $p_{0}$ is the prediction of the last observation. The prediction error is given by the absolute value on the difference between the forecast and the *t* observation. The variance $Var(E_{t})$ is calculated considering the prediction error at the prediction period *t* (i.e., the horizon of the estimation). In addition, the thresholds include a parameter *K*, from which use case operators can adjust the sensitivity of the upper and lower limits.

#### 3.3.4. Pattern Recognition Implementation

The instantiation of the framework considered at the performed experimentation assumes that the use case operators provide the collection of reference samples to be taken into account throughout the pattern recognition process. This implementation embraces the Attribute-Relation File Format (ARFF), Comma-Separated Values (CSV) or Packet Capture (pacp) feature descriptions in order to represent the reference datasets required for the construction of data mining models [77], in this way assuming their advantages, but also their drawbacks. As is the case on the Prediction component, a battery of pattern recognition and novelty detection methods is considered, which is summarized in Table 3. The decision of the best approach and calibration is driven by the results in terms of accuracy of a cross-validation test launched at training step.

#### 3.3.5. Knowledge Inference Implementation

It is well known that one of the classical problems with the software that implements Rete algorithms is the lack of interoperability with other high-level languages and complex data structures, such as class hierarchies, complex knowledge representations or abstract data. Nowadays there are few open source implementations with these capabilities, as is the case of Drools [87]. Given that its effectiveness has been continuously proved in European projects of different nature [88,89], Drools was implemented in the current instantiation of the proposed framework in order to manage the execution of the rule-based knowledge acquisition at the knowledge inference component. As highlighted by their authors, Drools is a Business Rules Management System (BRMS) solution that provides, among others, a core Business Rules Engine (BRE) and a modification of the original Rete algorithm adapted to Object-oriented scenarios which also bring solutions to optimization problems, such as rule priorization, concurrency execution of tasks, changes on rule execution modes, synchronization of events, different forms of metadata or sliding processing.

## 4. Experiments

The following describes the evaluation scenario, reference datasets and the use cases considered throughout the performed experimentation.

### 4.1. Evaluation Scenario

Since there are no collections of 5G network traffic, the performed experimentation is focused on the study of traffic traces gathered as public domain datasets, hence facilitating the replication of the obtained results. In particular, the evaluation scenario is focused on the study of real traffic monitored on high-speed Internet backbone links published at 2016 within the CAIDA anonymized Internet Traces Dataset [90]. With this purpose an illustrative use case is defined, which guides the knowledge acquisition framework to infer new facts related with the variations of the traffic volume monitored. It is important to highlight that since the dataset only provides raw data, it is not possible to corroborate the incidences discovered with those identified by their authors. However, such disadvantage is compensated by the fact that CAIDA is a well-known dataset with realistic information about current networks in the backbone, particularly in a data center. On the other hand, the SELFNET project is targeted to deal not only with similar, but with even more complex traffic samples collected in a telecommunications operator data center, where its instantiation and deployment is expected. Therefore the analysis of data center traffic is a scenario that the proposed framework (currently implemented as part of the SELFNET project) must address in a near future. Throughout the experimentation, this framework has been instantiated according to the orchestration strategy introduced by Barona et al. [54]. In this way, the analytic components act sequentially as sets of actions in the following order: pattern recognition, prediction, adaptive thresholding and knowledge inference. They are instantiated as described in section III: pattern recognition includes the battery of algorithms detailed in Table 3, prediction integrated the forecast methods summarized in Table 2, adaptive thresholding adapts the method published in [76], and knowledge inference imports the engine provided by Drools [87]. Both prediction and pattern recognition capabilities have been evaluated according to functional standardized methodologies. Firstly, the effectiveness of the forecast capabilities was tested adopting the M3-Competition scheme [67], in this way facilitating the comparison of the obtained results with previous publications. On the other hand, pattern recognition is validated based on the NSL-KDD dataset and the evaluation methodology proposed in [91]. As in the previous test, the results are contrasted with contributions that introduced similar features. Adaptive thresholding and knowledge inference implement well-known techniques previously considered in similar projects, so their effectiveness is assumed prior to the experimentation stage.

### 4.2. Reference Datasets

The performed experimentation applied three collections of reference data: NSL-KDD, M3-Competition and CAIDA’16. They are described below.

#### 4.2.1. NSL-KDD

NSL-KDD is a dataset suggested to solve some of the inherent problems of the KDD’99 dataset, which were reviewed by Tavallaee et al. in [91], among them: presence of redundant records in the training set, record duplication, or imbalance of the number of samples per group. Note that quoting their authors “this new version of the KDD data set still suffers from some of the problems discussed by McHugh [92] and may not be a perfect representative of existing real networks, because of the lack of public data sets for network-based IDSs, we believe it still can be applied as an effective benchmark data set”. Additionally, NSL-KDD authors analyzed the difficulty level of the samples in KDD’99, and according to the results, they proposed two different collections: KDDTrain+_20Percent KDD’9$9^{+}$) and KDDTest−21 (KDD’9$9^{-21}$), where the second includes records with difficulty level of 21 out of 21. It is important to note that according to Bhatia et al. [93], KDD’99 is one of the most referenced methodologies in the bibliography, and possibly the only one that presents a dataset of network security incidences with reliable labeling. The original KDD’99 collection was created for the competition KDD Cup, and it is based on the captures of traffic provided by the DARPA’98 dataset; in particular, legitimate (class *normal*, 97,277 (19.69%)) samples and the following simulated threats:

- Denial of Service attack (DoS): classes *back*, *land*, *neptune*, *pod*, *smurf* and *teardrop*; 391,458 (79.24%) instances.
- User to Root attack (U2R): classes *Buffer overfow*, *loadmodule*, *perl* and *rootkit*; 52 (0.01%) instances.
- Remote to Local attack (R2L); classes *Guess_passwd*, *ftp_write*, *imap*, *phf*, *multihop*, *warezmaster*, *warezclient* and *spy*; 1126 (0.23%) *instances*.
- Probing attacks: classes *satan*, *ipsweet*, *nmap* and *portsweep*; 4107 (0.83%) instances.

Their samples are characterized by 41 different features usually divided into three groups: basic features, traffic features and content features. The first group gathers all the attributes that can be extracted from a TCP/IP connection (e.g., duration, protocol, service, src_bytes, flag, etc.). On the other hand, the traffic features are computed with respect to a window interval, and describe host features (e.g., dst_host_count, dst_host_same_srv_rate, dst_host_serror_rate, etc.) and server features (e.g., srv_count, srv_serror_rate, diff_srv_rate, etc.). Finally, a group of features provides information able to unmask suspicious behaviors in the data portion, i.e., independent of the time period (e.g., root_shell, logged_in, hot, urgent, etc.). KDD’99 proposed as evaluation methodology to split the dataset into two groups: a 20% subset as training samples and the rest for testing. Note that NSL-KDD sanitized the original collection eliminating 78.05% of training samples (93.32% attack instances, 16.44% normal instances), and 75.15% of the test set (88.26% attack instances, 20.92% normal instances). Given that most of the discards where instances repeated in training and evaluation samples, the evaluation of classifiers with NSL-KDD displays considerably less precise results than KDD’99, posing much greater difficulty to the evaluated proposals. The SELFNET Pattern Recognition testbed adapts this scheme for evaluating the accuracy of the classification and novelty detection actions.

#### 4.2.2. M3 Competition

To the best of the author’s knowledge, there are not standardized methodologies to assess the effectiveness of forecasting algorithms on 5G environments; in fact, there are also no collections of samples of these monitoring scenarios. In view of this, the most reliable way of demonstrating the capacity of the SELFNET prediction framework is to evaluate it from general purpose methodologies adapted to time series prediction. Among them it is worth considering a well-known scheme such as the M3-Competition [67]. It provides a collection of 3003 time series categorized as: financial, industry, macroeconomics, microeconomics, demography and other. In order to ensure that every prediction method is able to process the proposed data, it was observed that time series have a minimum length of 14 observations for Yearly series (the median is 19 observations), 16 for Quarterly series (the median is 44 observations), 48 for Monthly time series (the median is 115 observations), and 60 for other series (the median is 63). Hence three blocks of data are clearly described: Yearly, Quarterly and Monthly. Note that all the time series are positive to avoid problems related with the various MAPE measures. If the original time series has negative values, they are replaced by zero. Table 4 displays the classification of these time series. In the original competition, the participants run their algorithms considering several prediction horizons (i.e., prediction periods): from t+1 to t+6 on Yearly data, from t+1 to t+8 for Quarterly data and from t+1 to t+18 for Monthly data. The dataset was evaluated according to five metrics: symmetric Mean Absolute Percentage Error (MAPE) or sMAPE, Average Ranking, median symmetric APE, Percentage Better, and median RAE (Relative Absolute Error). Among them, the sMAPE is the most frequent in the bibliography, bearing in mind both old and recent contributions. Because of this, sMAPE is the base of the performed experimentation.

#### 4.2.3. CAIDA Anonymized Internet Traces 2016

The Center for Applied Internet Data Analysis (CAIDA) has published the Anonymized Internet Traces 2016 Dataset [90], which contains traces obtained through the passive equinix-chicago monitor located at the Equinix [94] datacenter in Chicago. These traces represent real internet traffic samples used for research purposes. Moreover, it is important to bear in mind that all traces are anonymized, and their payload has been removed. Thereby the resultant pcap files store only layer 3 and layer 4 packet headers to be accounted when gathering network statistics. Traces are, in fact, an hour monitored traffic captured each month. Even when traffic traces are stored each month, current yearly CAIDA datasets are a collection of four Internet traffic trace (one per quarter).A one-hour traffic trace is split in several pcap files, each of them corresponding to a one-minute traffic. Currently, CAIDA 2016 dataset has published Internet traces captured at 21 January (Ds-January), 18 February (Ds-February), 17 March (Ds-March), and 6 April (Ds-April). All of them captured from 14:00:00 to 14:59:59.

The first part of the experimentation was conducted using a three-minutes sample of traffic traces extracted from Ds-Jan. They correspond to network data packets captured from 14:00:00 to 14:02:59. In order to measure traffic volume, a time series of 180 elements was constructed by accumulating the total number of bytes per second. At this stage of the research, it was not feasible to extend the length of the time series due to storage and parsing time limitations. Henceforth, this sample data is referred as CAIDA’16-sample.

In the second part of the experimentation, network traffic measures were gathered from the statistics files published by CAIDA. Each statistics file corresponds to a one-minute traffic observed in a one-hour dataset. Thereby, every dataset has 60 statistics files. Unlike the described CAIDA’16-sample, there was no need to parse pcap files since every one-minute statistics file provides the total number of transmitted bytes. This is exactly the same metric used in the first part of the experimentation, being the only difference the granularity. Consequently, four time series of 60 elements were constructed from Ds-Jan, Ds-Feb, Ds-Mar and Ds-Apr, being each element of the time series the observed traffic volume expressed in bytes per minute. Henceforth, the generated time series are referred as CAIDA’16-monthly.

### 4.3. Use Case: Detection of Anomalous Traffic Volume Variations

This use case goal is to infer if the observed network traffic volume presents an anomalous behavior. For this purpose, the knowledge inference framework components are instantiated, contributing to the generation of new facts that are used to evaluate the production rules configured to infer knowledge. The process is triggered once the novelty detection capabilities of the Pattern Recognition component identify a change in the network behavior. This task requires building a model of the normal traffic behavior, which is created by assuming the first observations as reference samples and their following six attributes: Euclidean, Squared $X^{2}$, Canberra, Pearson, Bhattacharyya and Divergence distances between the last two observations. The use of these metrics in network anomaly detection is detailed reviewed in [95].

Provided by the generated facts about possible anomalous traffic pattern, the Prediction component calculates the forecasting values for the time series considering 1, 5 and 10 time horizons, and the results are also inserted in the working memory. With the forecasted metrics, the Adaptive Thresholding component deduces the prediction intervals (PI) for each observation, registering them in the working memory as new acquired facts. The upper and lower thresholds are computed upon the forecasting error. Previously generated facts about abnormal traffic patterns, forecasting values and thresholds allow the Inference Engine to deduce the existence of anomalous traffic volume variations when two conditions are met: traffic volume has been labeled as abnormal and the observation is either exceeding the upper prediction interval or below the lower bound. Note that combining both of them allows considering the presence of outliers regarding the general traits of the behavior observed in the monitored environment, as well as unexpected variations from the latest observations. In this way, the incidents will be reported with greater certainty about their nature.

## 5. Results

The following describes the results obtained when analyzing the aforementioned datasets.

### 5.1. Prediction Capabilities Evaluation

The M3 dataset described in the previous section led to the evaluation of the framework under different time series; being Yearly, Monthly, Quarterly and Others the time series classifications as described in Table 4. The results of the evaluated forecasting methods are shown in Table 5, Table 6, Table 7 and Table 8. For each method, the sMAPE value for a given forecasting horizon (t+1 up to t+18) is in fact the mean of the sMAPE values obtained for the same forecasting horizon in a set of time series (#Obs) with the same data nature. Yearly data has been evaluated under the proposed framework and their results are detailed in Table 5. The obtained mean sMAPE values computed over 645 time series range from 6.6 to 9.4, thus, exposing a better accuracy for all the evaluated forecasting horizons (t+1 to t+6) compared to the other forecasting algorithms used in the M3 competition. Consequently, an average sMAPE of 7.1 computed for the 1 to 4 horizons, and a 7.7 value for the 1 to 6 horizons, expose also an overall better accuracy in comparison with the M3 methods, which values range from 13.65 to 21.59. Quarterly data results are shown in Table 6. The mean sMAPE values were computed by the proposed framework over 756 time series, and they range from 4.4 to 5.2, thus, exposing a better accuracy for most of the evaluated forecasting horizons (t+1 to t+8), being t+1 the only case where the framework does not show the best performance compared to the other M3 forecasting algorithms. However, the average sMAPE values of 6 for the 1 to 4, 4.9 for the 1 to 6, and 4.8 for the 1 to 8 forecasting horizons shown a better accuracy, particularly when the horizon is incremented. The average sMAPE for the existing methods are in fact ranging from 7 to 10.96 in any case. Monthly data has also been evaluated under the framework, presenting their results in Table 7. The obtained mean sMAPE values computed over 1428 time series range from 9.6 to 12.7, exposing again a better accuracy for most of the evaluated forecasting horizons (t+1 to t+18) with values ranging from 9.6 to 12.7, being t+2 and t+4 the only cases where the framework has a slightly less performance of −0.5 and −0.1 for t+2 and t+4, respectively, compared with the mean SMAPE obtained by other algorithms with values ranging from 10.7 to 24.3, considering all the forecasting horizons. Hence, the average sMAPE values also show the best accuracy for the proposed framework, with values ranging from 11.1 to 11.6, being only the average sMAPE of 11.6 for the 1 to 4 horizons slightly bigger than the lowest one in this category, obtained by Theta (11.54). The remaining M3 average sMAPE values computed for the 1 to 6, 1 to 8, 1 to 12, 1 to 15 and 1 to 18 forecasting horizons range from 11.54 to 18.4 in any case. Therefore, this overall results exposed the best accuracy with Monthly data. It is worth mentioning that this set of time series are the longest used in the competition (with a mean of 115 observations). Finally, Other data has also been evaluated following the same approach used for Quarterly data, but with 174 time series (see Table 8). As compared to the preceding time series categories (Yearly, Quarterly and Monthly), in this case the results were significantly better, except for the t+1 horizon where the mean sMAPE obtained by this proposal was 1.8 compared with the minimum value of 1.6 obtained by the Autobox 2 method. The remaining forecasting horizons shown a value ranging from 1.5 to 2.4, exposing an increasing accuracy as long as the forecasting horizon grows. In consequence, the average sMAPE values for the 1 to 4, 1 to 6 and 1 to 8 horizons show also better results when the framework performs the forecasting.

### 5.2. Pattern Recognition Capabilities Evaluation

The results obtained for the different classifiers at pattern recognition actions considering NSL-KDD’9$9^{+}$ are summarized in Table 9, and the results with NSL-KDD’9$9^{-21}$ are displayed in Table 10. On the other hand, Table 11 compares the effectiveness of the SELFNET Pattern Recognition set of actions with some of the most relevant proposals in the bibliography; in particular, those reviewed by Ashfaq et al. [96]. This publication was released at early 2017 and discusses the effectiveness of most of the latest proposals for intrusion detection that assumed the NSL-KDD’99 evaluation methodology, in this way assuming as principal classification criterion the accuracy they proved. In the case of the subset of samples NSL-KDD’9$9^{+}$, the best classifier in SELFNET was Adaptive Boosting with 82.2% accuracy. This result is close to the best accuracy in the reviewed bibliography (84.12%), where the clustering approach introduced by Hernández-Pereira [97] was applied on flag and service features of the dataset, combined with the fuzziness based semi-supervised learning approach proposed by Ashfaq et al. Bearing in mind that in this experiment the SELFNET Pattern Recognition framework did not use data preprocessing capabilities (unlike in the aforementioned publication), it is possible to conclude that SELFNET effectiveness is sufficient for the next experiments, hence leaving preprocessing for future implementations. In the second test, the subset of samples NSL-KDD’9$9^{-21}$ was considered. The best configuration of the SELFNET Pattern Recognition framework achieved 89.9% accuracy when executed with generation of synthetic samples. The average accuracy on the latest publications is 60.3%; in particular, the best classifier tested by Ashfaq et al. demonstrated 68.2% accuracy when considering Adaptive Boosting and the previously described preprocessing. Again, it is possible consider that the achieved effectiveness is enough to validate its effectiveness.

### 5.3. Use Case Evaluation

The following sections describe the two experiments carried on upon the CAIDA’16 reference dataset, analyzed under different levels of data granularity for each: per second and per minute.

#### 5.3.1. Experiment 1: CAIDA’16-Sample

**Figure 3:**
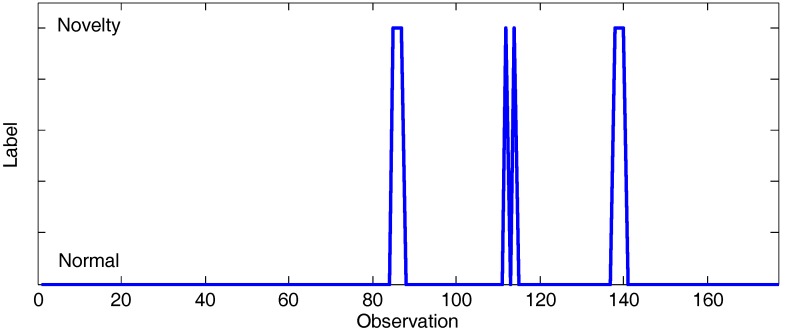
Discordant observations at novelty detection for CAIDA’16-sample.

**Figure 4:**
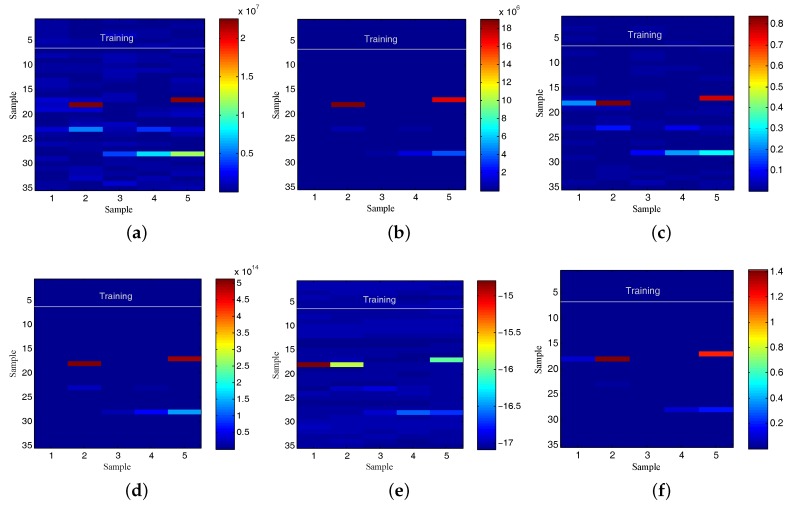
Metric variations on samples. (**a**) Euclidean; (**b**) Quadratic $X^{2}$; (**c**) Canberra; (**d**) Pearson; (**e**) Bhattacharyya; (**f**) Divergence.

The first step on the CAIDA traffic volume analysis according to the aforementioned use case is novelty detection. With this purpose, the first 35 observations on the monitored environment are considered as reference samples for building the normal network usage model. The evaluation of the model demonstrated 91.4894% accuracy when tested via cross-validation. The best selected pattern recognition setting was the combination of generating synthetic data as counterexample [86] and its analysis with Bootstrap Aggregation [81] based on decision stump [78]. Discordant traffic volume values were monitored at observations 86–88 (21 January 2016 14:01:25 to 14:01:29), 113 (21 January 2016 14:01:54), 115 (21 January 2016 14:01:56), 139–141 (21 January 2016 14:02:20 to 14:02:23). Figure 3 summarizes the anomalous observations discovered. The impact of the six attributes taken into account is illustrated in Figure 4. As can be observed, each of them highlights the fact that at the aforementioned observations on the traffic volume, there is a discordant with the reference data.

**Figure 5:**
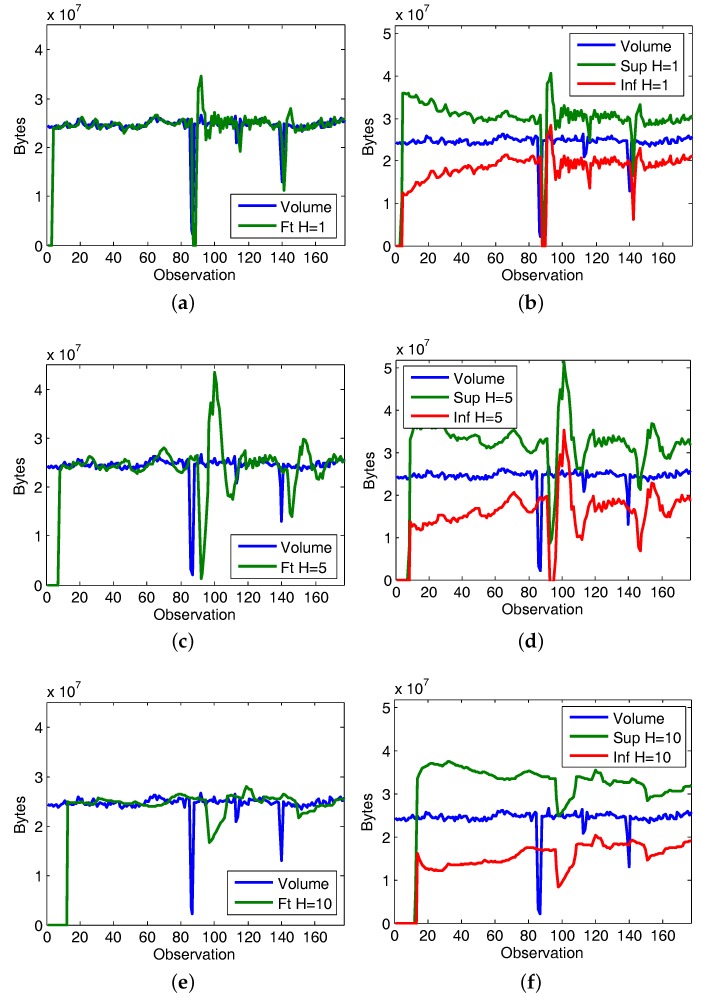
Evolution of prediction and adaptive thresholding on CAIDA’16 sample. (**a**) Forecast *H* = 1; (**b**) Threhsolding *H* = 1; (**c**) Forecast *H* = 5; (**d**) Threhsolding *H* = 5; (**e**) Forecast *H* = 10; (**f**) Threhsolding *H* = 10.

The next knowledge acquisition step is to infer new facts from predictions. The obtained results are summarized in Table 12, and Figure 5 illustrates the evolution of the predictions for horizon 1 (Figure 5a), horizon 5 (Figure 5c) and horizon 10 (Figure 5e). From them it is easy deduce that the higher horizon, the higher forecast error. On the other hand, their different adaptive thresholds are shown in Figure 5b,d,f. The thresholds provide greater margin of error when the forecasting error is higher. Because of this, the selection of an appropriate horizon plays an essential role in the use case effectiveness, since it conditions the level of restriction on which operates the knowledge acquisition framework.

**Figure 6:**
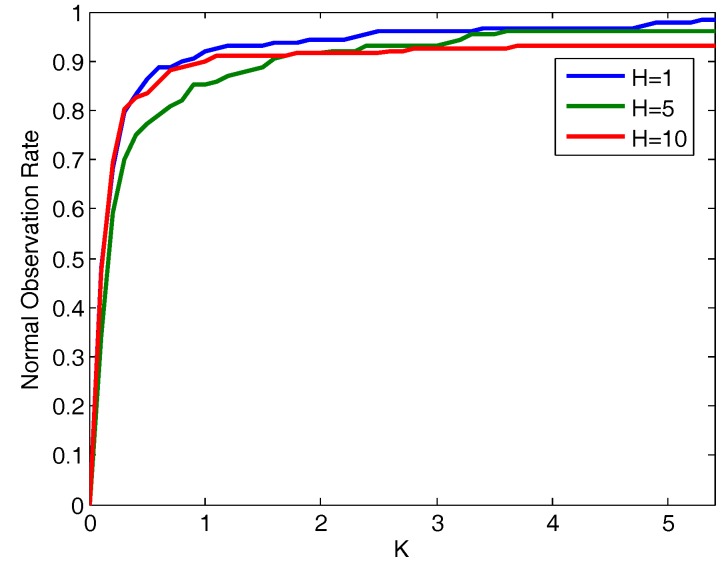
Normal observation rate when varying *K*.

Another aspect to keep in mind is the impact of the *K* adjustment value at the decisions taken. This parameter regulates the restraint of the adaptive thresholds. Figure 6 illustrated the variation of the ratio of observations tagged as normal when modifying *K*. Regardless of the prediction horizon, when *K* shows lower values the number of observations labeled as unexpected is higher; hence the level of restriction on which the framework operates is higher. Conversely, as *K* grows the normal labeling rate increases, in this way overlooking situations that in the previous cases were considered discordant.

**Figure 7:**
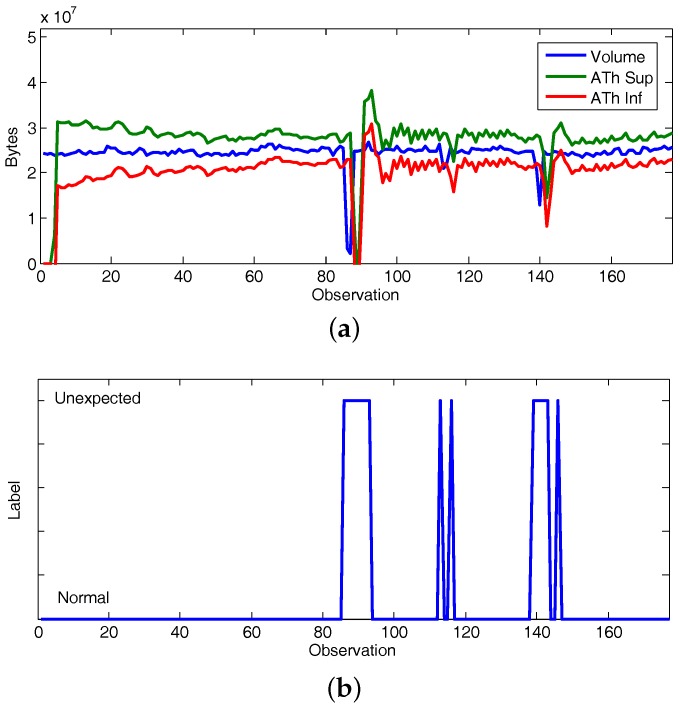
Thresholds and Unexpected traffic on CAIDA’16-sample. (**a**) Traffic volume variation on CAIDA’16-sample; (**b**) Unexpected observations on CAIDA’16-sample.

Finally, letting a forecasting horizon of 1 observation and *K* = 1, Figure 7a illustrated the traffic volume evolution on CAIDA’16-sample and the adaptive thresholds inferred at the proposed framework. Figure 7b summarizes the unexpected observations discovered, which provide the rest of the information required to produce conclusions (symptoms). Unexpected traffic volumes occur at observations 86–93 (21 January 2016 14:01:25 to 14:01:34), 113 (21 January2016 14:01:54), 115 (21 January 2016 14:01:56), 139–143 (21 January 2016 14:02:20 to 14:02:25) and 146 (21 January 2016 14:02:25). By contrasting Figure 3 and Figure 7b it is possible deduce when the knowledge acquisition instantiation of the proposed framework infers symptoms as final facts related with the implemented use case (i.e., observations tagged as “suspicious”). It takes place each time a novelty behavior is discovered and the traffic volume is unexpected, which occurs at observations 86–88 (21 January 2016 14:01:25 to 14:01:29), 113 (21 January 2016 14:01:54), 115 (21 January 2016 14:01:56), 139–141 (21 January 2016 14:02:20 to 14:02:23).Suspicious variations on the volume of the monitored data are reported to the decision-making sub-layer, where the countermeasures to be deployed are planned and orchestrated.

#### 5.3.2. Experiment 2: CAIDA’16-monthly

**Figure 8:**
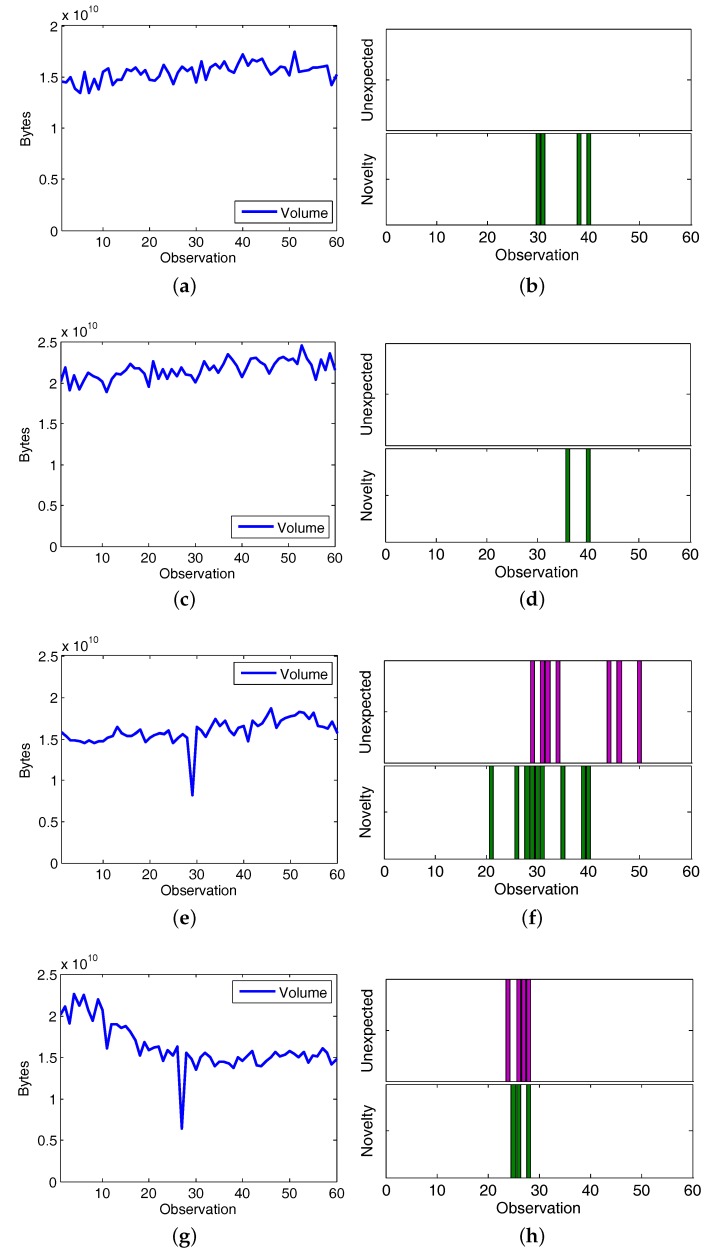
Evolution of observations, thresholding and novelty detection on CAIDA’16 monthly. (**a**) Observations (January); (**b**) Symptoms (January); (**c**) Observations (February); (**d**) Symptoms (February); (**e**) Observations (March); (**f**) Symptoms (March); (**g**) Observations (April); (**h**) Symptoms (April).

This experiment was performed upon CAIDA’16-monthly data, by following the same approach conducted in the previous section. Since CAIDA’16-monthly is composed by four time series (traffic traces collected at January, February, March and April), they are individually analyzed by the framework. Being novelty detection and forecasting the set of actions in the knowledge acquisition process, the conclusions deduced by the framework are summarized in Figure 8. Observations are shown in Figure 8a,c,e,g; and the comparison between novelty detection and unexpected traffic detected at each monthly dataset are plotted in Figure 8b,d,f,h, which are the required facts to produce symptoms.

When analyzing CAIDA’16 at January and February it can be concluded that even when discordant observations are detected in the novelty detection stage, unexpected traffic volumes are not observed in any of the time series. Thereby, there are no anomalous traffic volume variations reported by the framework. It is explained due to the required conditions (novelty and unexpected traffic) do not occur simultaneously at any observation.

On the other hand, March and April CAIDA’16 datasets detected both unexpected and discordant traffic volume observations in the analyzed time series. By contrasting the traffic labeled as “unexpected” with the novelty detection results at the March dataset (Figure 8f), the inference of anomalous traffic symptoms take place at observations 29 (17 March 2016 14:28) and 31 (17 March 2016 14:30). Likewise, at the April dataset (Figure 8h), symptoms are inferred at observations 29 (6 April 2016 14:28), 31 (6 April 2016 14:30), and 32 (6 April 2016 14:31); where network traffic is simultaneously considered “unexpected” and “fluctuant”, so the two required conditions to trigger the “suspicious” traffic symptoms are met.

### 5.4. Discussion

To validate the current reasoning framework, the architecture was instantiated adapted to the introduced design. It allowed the acquisition of facts and generation of knowledge in the analysis of a real use case. The instanced framework demonstrated the interaction of the components involved to deduce conclusions regarding anomalous network traffic volumes. The different steps followed in the knowledge generation process were also explained in the paper, being the prediction and patter recognition actions more extensively detailed since they exposed the more complex efforts. The suitable integration of the instantiated components validates the applicability of the proposed framework, and its effectiveness for the evaluation of a use case.

In order to determine the accuracy of the prediction and patter recognition capabilities, the individual evaluation of each component has been performed. To this end, publicly available datasets were taken as a baseline. On the one hand, accuracy was measured in the prediction module, in comparison with the forecasting errors extracted from the M3-competition dataset. The prediction capabilities have proven acceptable accuracy levels when contrasted with other methods in the majority of forecasted scenarios of the M3-Competition. It was accomplished by the efficient selection of the right prediction algorithms included in the module, where parameter calibration played an important role when minimizing the forecasting error. Consequently, the decision of the best method has been determined by the evaluation of the lowest prediction error. On the other hand, the accuracy of patter recognition capabilities was also evaluated. With this purpose, the NSL-KDD’9$9^{+}$ and NSL-KDD’9$9^{-21}$ datasets were taken to measure the classification accuracy. In both scenarios, the instantiated patter recognition module exposed effective accuracy levels compared with similar research approaches documented in recent publications. It is important to remark the experimental rigor at this stage since the evaluated use case was strongly dependent on the effectiveness of both prediction and patter recognition features.

With the instantiated framework, the CAIDA’16 dataset allowed to analyze anomalous traffic on real internet traces. It was proven that the implemented reasoning and knowledge acquisition capabilities facilitate the comprehension of the network status. Traffic analysis was performed at different granularity levels exposing the effectiveness of the framework to deduce symptoms of anomalous traffic. The prediction component obtained precise estimations of the traffic volume at different forecasting horizons. It led to the accurate construction of adaptive thresholds that determined the discovery of unexpected traffic volumes. Moreover, the pattern recognition module performed novelty detection actions to find fluctuations of the monitored data. The generated model was also effective when identifying network traffic fluctuations. Hence, the outcomes of the prediction and patter recognition modules allowed an overall accuracy of the whole use case.

Throughout the development of the proposal several design principles and restrictions were introduced. They were carefully taken into account not only in the design but also in the instantiation of the framework. Use case onboarding is supported by the insertion of procedural knowledge in the framework. Reference datasets were also applied for evaluation purposes, but it must be recalled that the validity of the results is tightly dependent on the datasets accuracy as show in this paper. The framework also considered a stationary environment in the experimentation, hence demonstrating its effectiveness. The main goal of this proposal is to generate useful knowledge following an orchestrated information flow, which was accomplished by the definition and instantiation of this framework.

Finally, it is important to bear in mind that the framework itself is a modular architecture which separates analytical processes into different sets of actions coordinated by an orchestration strategy. These processes are completely independent and only share their input and output data, hence accommodating vertical and horizontal scalability, execution of tasks in concurrence and their deployment on different machines. Likewise, each set of actions can deploy different analytical tools, and so far, it was not encountered any incompatibility involving the incorporation of additional capabilities such as big data mechanisms. These criteria are compliant with the most recent network “softwarization” trends grounding the development of 5G networks.

## 6. Conclusions

This paper introduced a reasoning and knowledge acquisition framework adapted to support analytic actions on 5G monitoring environments toward providing self-organized autonomic networks. Because of this, the framework assumes the design principles of the new generation networks and the technologies they implement, being instantiated as part of the solutions brought by the SELFNET Horizon 2020 project. This involves the implementation of different techniques and algorithms based, inter alia, on pattern recognition, prediction, adaptive thresholding or knowledge inference; which are orchestrated as a modular architecture, easily expandable and scalable, adapted to the large volume of information flowing through these challenging scenarios.

In order to facilitate the understanding of the conducted research, an instantiation of this framework has been performed. For this purpose, every component has been equipped with very basic capabilities, which has led to the implementation of well-known data mining algorithms and machine learning schemes within each of them. The experimentation has focused on two fundamental aspects: demonstrating that each component operates properly and testing the potential of the proposal in a real use case. At the first stage, the functional standards NSL-KDD and M3-competition have been considered. The obtained results corroborated the efficacy of the deployed components by comparison with similar proposals. On the other hand, the defined use case allowed the acquisition of knowledge related to variations of traffic volume on the monitoring environment. This has been tested with real traffic, in particular with traces provided by the CAIDA’16 collection. The results demonstrated that regardless of the granularity in which the observed information is studied, the proposal is able of generating useful information for the management of the occurred incidents.

Notwithstanding as part of the contributions aimed on the development of 5G networks, the proposal rises new research lines. The clearest of them is to delve into how the knowledge acquisition framework can be instantiated in order to face the challenges posed by the different use cases. These may have very different requirements; for example, a use case focused on bandwidth optimization may require information that facilitates deciding proactive actions, and therefore must be primarily based on prediction; but the reactive responses have greater impact on mitigating threats such as botnets or denial of service attacks. Other interesting topics, such as inclusion of data protection policies or the communication ways between the proposed framework and the rest of network components (protocols, interfaces, etc.), have not been detailed throughout the article, in this way postponing their development for future work.

## Figures and Tables

**Table 1:** Summary of the instantiated initial knowledge and notifications.

**Initial Factual Knowledge**	
**Element**	**Description**
Object	Traffic volume monitored per sensor ($V_{t}$).
Acquisition	Via ADB.
**Procedural Knowledge**	
**Component**	**Behavior**
Prediction	Forecast traffic volume ($V_{t}$) given a certain prediction horizon.
Adaptive Thresholding	Construction of decision thresholds from forecasted metrics.
Pattern Recognition	Novelty detection based on several distances and similarity metrics related with $V_{t}$ and $V_{t-1}$.
Knowledge Inference	It is deduced that if $V_{t}$ observations exceed adaptive thresholds, they are *unexpected*. If $V_{t}$ observations are considered novelties, it is deduced that they are *fluctuations*. If $V_{t}$ is *unexpected* and *fluctuation* then it is *suspicious*.
Notification	Reports *suspicious* events are reported via message broker software.

**Table 2:** Battery of forecasting algorithms.

Method	Type
Cumulative Moving Average (CMA) [68]	Moving Average
Simple Moving Average (SMA) [69]	Moving Average
Double Moving Average (DMA) [69]	Moving Average
Weighted Moving Average (WMA) [70]	Moving Average
Simple Exponential Smoothing (EMA) [71]	Moving Average
Double Exponential Moving Average (DEMA) [72]	Moving Average
Triple Exponential Moving Average (TEMA) [69]	Moving Average
Simple Exponential Smoothing (SES) [73]	Smoothing
Double Exponential Smoothing (DES) [74]	Smoothing
Triple Exponential Smoothing (TES) [75]	Smoothing

**Table 3:** Battery of pattern recognition algorithms.

Method	Action
Decision Stump [78]	Classification
Reducing Error Pruning Tree [79]	Classification
Random Forest [80]	Classification
Bootstrap Aggregation [81]	Classification
Adaptive Boosting [82]	Classification
Bayesian Network [83]	Classification
Naive Bayes [84]	Classification and Novelty detection
Support Vector Machines (SVM) [85]	Classification and Novelty detection
Generation of synthetic data + Bootstrap Aggregation [86]	Novelty detection

**Table 4:** Time series categories and attributes of the M3 dataset.

	Micro	Industry	Macro	Finance	Demographic	Other	Total
Year	146	102	83	58	245	11	645
Quart.	204	83	336	76	57		756
Month	474	334	312	145	111	141	1428
Other	4			29		141	174
Total	828	519	731	308	413	204	3003

**Table 5:** SMAPE on M3-Competition for Yearly data.

Method	Forecasting Horizon						Average		#Obs
	*t* + 1	*t* + 2	*t* + 3	*t* + 4	*t* + 5	*t* + 6	1 to 4	1 to 6	
Naive	8.5	13.2	17.8	19.9	23	24.9	14.85	17.88	645
Single	8.5	13.3	17.6	19.8	22.8	24.8	14.82	17.82	645
Holt	8.3	13.7	19	22	25.2	27.3	15.77	19.27	645
Dampen	8	12.4	17	19.3	22.3	24	14.19	17.18	645
Winter	8.3	13.7	19	20	25.2	27.3	15.77	19.27	645
Comb S-H-D	7.9	12.4	16.9	24.1	22.2	23.7	14.11	17.07	645
B-J automatic	8.6	13	17.5	18.2	22.8	24.5	14.78	17.73	645
Autobox 1	10.1	15.2	20.8	22.5	28.1	31.2	17.57	21.59	645
Autobox 2	8	12.2	16.2	19	21.2	23.3	13.65	16.52	645
Autobox 3	10.7	15.1	20	20.4	25.7	28.1	17.09	20.36	645
Robust-Trend	7.6	11.8	16.6	20.3	22.1	23.5	13.75	16.78	645
ARARMA	9	13.4	17.9	19.1	23.8	25.7	15.17	18.36	645
Automat ANN	9.2	13.2	17.5	19.7	23.2	25.4	15.04	18.13	645
Flores/Pearce 1	8.4	12.5	16.9	19.1	22.2	24.2	14.22	17.21	645
Flores/Peace 2	10.3	13.6	17.6	19.7	21.9	23.9	15.31	17.84	645
PP-autocast	8	12.3	16.9	19.1	22.1	23.9	14.08	17.05	645
ForecastPro	8.3	12.2	16.8	19.3	22.2	24.1	14.15	17.14	645
SmartFcs	9.5	13	17.5	19.9	22.1	24.1	14.95	17.68	645
Theta-sm	8	12.6	17.5	20.2	13.4	25.4	14.6	17.87	645
Theta	8	12.2	16.7	19.2	21.7	23.6	14.02	16.9	645
RBF	8.2	12.1	16.4	18.3	20.8	22.7	13.75	16.42	645
ForecastX	8.6	12.4	16.1	18.2	21	22.7	13.8	16.48	645
**This proposal**	**6.9**	**6.6**	**7.6**	**7.2**	**8.5**	**9.4**	**7.1**	**7.7**	**645**

**Table 6:** SMAPE on M3-Competition for Quarterly data.

Method	Forecasting Horizon							Average			#Obs
	*t* + 1	*t* + 2	*t* + 3	*t* + 4	*t* + 5	*t* + 6	*t* + 8	1 to 4	1 to 6	1 to 8	
Naive	5.4	7.4	8.1	9.2	10.4	12.4	13.7	7.55	8.82	9.95	756
Single	5.3	7.2	7.8	9.2	10.2	12	13.4	7.38	8.63	9.72	756
Holt	5	6.9	8.3	10.4	11.5	13.1	15.6	7.67	9.21	10.67	756
Dampen	5.1	6.8	7.7	9.1	9.7	11.3	12.8	7.18	8.29	9.33	756
Winter	5	7.1	8.3	10.2	11.4	13.2	15.3	7.65	9.21	10.61	756
Comb S-H-D	5	6.7	7.5	8.9	9.7	11.2	12.8	7.03	8.16	9.22	756
B-J automatic	5.5	7.4	8.4	9.9	10.9	12.5	14.2	7.79	9.1	10.26	756
Autobox 1	5.4	7.3	8.7	10.4	11.6	13.7	15.7	7.95	9.52	10.96	756
Autobox 2	5.7	7.5	8.1	9.6	10.4	12.1	13.4	7.73	8.89	9.9	756
Autobox 3	5.5	7.5	8.8	10.7	11.8	13.4	15.4	8.1	9.6	10.93	756
Robust-Trend	5.7	7.7	8.2	8.9	10.5	12.2	12.7	7.63	8.86	9.79	756
ARARMA	5.7	7.7	8.6	9.8	10.6	12.2	13.5	7.96	9.09	10.12	756
Automat ANN	5.5	7.6	8.3	9.8	10.9	12.5	14.1	7.8	9.1	10.2	756
Flores/Pearce 1	5.3	7	8	9.7	10.6	12.2	13.8	7.48	8.78	9.95	756
Flores/Peace 2	6.7	8.5	9	10	10.8	12.2	13.5	8.57	9.54	10.43	756
PP-autocast	4.8	6.6	7.8	9.3	9.9	11.3	13	7.12	8.28	9.36	756
ForecastPro	4.9	6.8	7.9	9.6	10.5	11.9	13.9	7.28	8.57	9.77	756
SmartFcs	5.9	7.7	8.6	10	10.7	12.2	13.5	8.02	9.16	10.15	756
Theta-sm	7.7	8.9	9.1	9.7	10.2	11.3	12.1	8.86	9.49	10.07	756
Theta	5	6.7	7.4	8.8	9.4	10.9	12	7	8.04	8.96	756
RBF	5.7	7.4	8.3	9.3	9.9	11.4	12.6	7.69	8.67	9.57	756
ForecastX	4.8	6.7	7.7	9.2	10	11.6	13.6	7.12	8.35	9.54	756
AAM1	5.5	7.3	8.4	9.7	10.9	12.5	13.8	7.71	9.05	10.16	756
AAM2	5.5	7.3	8.4	9.9	11.1	12.7	14	7.75	9.13	10.26	756
**This proposal**	**5.3**	**5.2**	**4.5**	**4.7**	**4.4**	**4.8**	**4.9**	**6.0**	**4.9**	**4.8**	**756**

**Table 7:** SMAPE on M3-Competition for Monthly data.

Method	Forecasting Horizon										Average						#Obs
	*t* + 1	*t* + 2	*t* + 3	*t* + 4	*t* + 5	*t* + 6	*t* + 8	*t* + 12	*t* + 15	*t* + 18	1 to 4	1 to 6	1 to 8	1 to 12	1 to 15	1 to 18	
Naive	15	13.5	15.7	17	14.9	14.7	15.6	15	19.3	20.47	15.3	15.13	15.29	15.57	16.18	16.91	1428
Single	13	12.1	12.1	15.1	13.5	13.1	13.8	14.5	18.3	19.4	13.53	13.44	13.6	13.83	14.51	15.32	1428
Holt	12.2	11.6	13.4	14.6	13.6	13.3	13.7	14.8	18.8	20.2	12.95	13.11	13.33	13.77	15.51	15.36	1428
Dampen	11.9	11.4	13	14.2	12.9	12.6	13	13.9	17.5	18.9	12.63	12.67	12.85	13.1	13.77	14.59	1428
Winter	12.5	11.7	13.7	14.7	13.6	13.4	14.1	14.6	18.9	20.2	13.17	13.28	13.52	13.88	14.62	15.44	1428
Comb S-H-D	12.3	11.5	13.2	14.3	12.9	12.5	13	13.6	17.3	18.3	12.83	12.79	12.92	13.11	13.75	14.48	1428
B-J automatic	12.3	11.4	12.8	14.3	12.7	12.6	13	14.1	17.8	19.3	12.78	12.74	12.89	13.21	13.96	14.81	1428
Autobox 1	13	12.2	13	14.5	14.1	13.4	14.3	15.4	19.1	20.4	13.27	13.42	13.71	14.1	14.93	15.83	1428
Autobox 2	13.1	12.1	13.5	15.3	13.3	13.8	13.9	15.2	18.2	19.9	13.51	13.52	13.76	14.16	14.86	15.69	1428
Autobox 3	12.3	12.3	13	14.4	14.6	14.2	14.8	16.1	19.2	21.2	12.99	13.47	13.89	14.43	15.2	16.18	1428
Robust-Trend	15.3	13.8	15.5	17	15.3	15.6	17.4	17.5	22.2	24.3	15.39	15.42	15.89	16.58	17.47	18.4	1428
ARARMA	13.1	12.4	13.4	14.9	13.7	14.2	15	15.2	18.5	20.3	13.42	13.59	14	14.41	15.08	15.84	1428
Automat ANN	11.6	11.6	12	14.1	12.2	13.9	13.8	14.6	17.3	19.6	12.31	12.55	12.92	13.42	14.13	14.93	1428
Flores/Pearce 1	12.4	12.3	14.2	16.1	14.6	14	14.6	14.4	19.1	20.8	13.74	13.93	14.22	14.29	15.02	15.96	1428
Flores/Peace 2	12.6	12.1	13.7	14.7	13.2	12.9	13.4	14.4	18.2	19.9	13.26	13.21	13.33	13.53	14.31	15.17	1428
PP-autocast	12.7	11.7	13.3	14..3	13.2	13.4	14	14.3	17.7	19.6	13.02	13.11	13.37	13.72	14.36	15.15	1428
ForecastPro	11.5	10.7	11.7	12.9	11.8	12.3	12.6	13.2	16.4	18.3	11.72	11.82	12.06	12.46	13.09	13.86	1428
SmartFcs	11.6	11.2	12.2	13.6	13.1	13.7	13.5	14.9	18	19.4	12.16	12.58	12.9	13.51	14.22	15.03	1428
Theta-sm	12.6	12.9	13.2	13.7	13.4	13.3	13.7	14	16.2	18.3	13.1	13.2	13.44	13.65	14.09	14.66	1428
Theta	11.2	10.7	11.8	12.4	12.2	12.4	12.7	13.2	16.2	18.2	11.54	11.8	12.3	12.5	13.11	13.85	1428
RBF	13.7	12.3	13.7	14.3	12.3	12.8	13.5	14.1	17.3	17.8	13.49	13.18	13.4	13.67	14.21	14.77	1428
ForecastX	11.6	11.2	12.6	14	12.4	12.2	12.8	13.9	17.8	18.7	12.32	12.31	12.46	12.83	13.6	14.45	1428
AAM1	12	12.3	12.7	14.1	14	14	14.3	14.9	18	20.4	12.8	13.2	13.63	14.05	14.78	15.69	1428
AAM2	12.3	12.4	12.9	14.4	14.3	14.2	14.5	15.1	18.4	20.7	13.03	13.45	13.87	14.25	15.01	15.93	1428
**This proposal**	**11.0**	**11.2**	**11.7**	**12.5**	**11.6**	**11.4**	**10.6**	**9.6**	**11**	**12.7**	**11.6**	**11.6**	**11.4**	**11.1**	**11.2**	**11.4**	**1428**

**Table 8:** SMAPE on M3-Competition for other data.

Method	Forecasting Horizon							Average			#Obs
	t+1	t+2	t+3	t+4	t+5	t+6	t+8	1 to 4	1 to 6	1 to 8	
Naive	2.2	3.6	5.4	6.3	7.8	7.6	9.2	4.38	5.49	6.3	174
Single	2.1	3.6	5.4	6.3	7.8	7.6	9.2	4.36	5.48	6.29	174
Holt	1.9	2.9	3.9	4.7	5.7	5.6	7.2	3.32	4.13	4.81	174
Dampen	1.8	2.7	3.9	4.7	5.8	5.4	6.6	3.28	4.06	4.61	174
Winter	1.9	2.9	3.9	4.7	5.8	5.6	7.2	3.32	4.13	4.81	174
Comb S-H-D	1.8	2.8	4.1	4.7	5.8	5.3	6.2	3.36	4.09	4.56	174
B-J automatic	1.8	3	4.5	4.9	6.1	6.1	7.5	3.52	4.38	5.06	174
Autobox 1	2.4	3.3	4.4	4.9	5.8	5.4	6.9	3.76	4.38	4.93	174
Autobox 2	1.6	2.9	4	4.3	5.3	5.1	6.4	3.19	3.86	4.41	174
Autobox 3	1.9	3.2	4.1	4.4	5.5	5.5	7	3.39	4.09	4.71	174
Robust-Trend	1.9	2.8	3.9	4.7	5.7	5.4	6.4	3.32	4.07	4.58	174
ARARMA	1.7	2.7	4	4.4	5.5	5.1	6	3.17	3.87	4.38	174
Automat ANN	1.7	2.9	4	4.5	5.7	5.7	7.4	3.26	4.07	4.8	174
Flores/Pearce 1	2.1	3.2	4.3	5.2	6.2	5.8	7.3	3.71	4.47	5.09	174
Flores/Peace 2	2.3	2.9	4.3	5.1	6.2	5.7	6.5	3.67	7.73	4.89	174
PP-autocast	1.8	2.7	4	4.7	5.8	5.4	6.6	3.29	4.07	4.62	174
ForecastPro	1.9	3	4	4.4	5.4	5.4	6.7	3.31	4	4.6	174
SmartFcs	2.5	3.3	4.3	4.7	5.8	5.5	6.7	3.68	4.33	4.86	174
Theta-sm	2.3	3.2	4.3	4.8	6	5.6	6.9	3.66	4.37	4.93	174
Theta	1.8	2.7	3.8	4.5	5.6	5.2	6.1	3.2	3.93	4.41	174
RBF	2.7	3.8	5.2	5.8	6.9	6.3	7.3	4.38	5.12	5.6	174
ForecastX	2.1	3.1	4.1	4.4	5.6	5.4	6.5	3.42	4.1	4.64	174
**This proposal**	**1.8**	**2.3**	**2.2**	**2.0**	**2.3**	**1.5**	**2.4**	**2.1**	**2.0**	**2.0**	**174**

**Table 9:** Results when analyzing NSL-KDD’9$9^{+}$.

Classifier	Class	TPR	FPR	Precision	Recall	F-Measure	MCC	AUC	PRC Area	Accuracy
Decision Stump	Normal	0.955	0.731	0.695	0.955	0.804	0.642	0.819	0.683	0.799
	Anomaly	0.683	0.045	0.952	0.683	0.795	0.642	0.819	0.831	
	Average	0.8	0.162	0.841	0.8	0.8	0.642	0.819	0.767	
RepTree	Normal	0.909	0.256	0.729	0.909	0.809	0.649	0.822	0.721	0.815
	Anomaly	0.744	0.091	0.915	0.744	0.821	0.649	0.822	0.858	
	Average	0.815	0.162	0.835	0.815	0.816	0.649	0.822	0.799	
Random Forest	Normal	0.973	0.323	0.695	0.973	0.811	0.658	0.959	0.947	0.803
	Anomaly	0.677	0.027	0.971	0.677	0.798	0.658	0.959	0.961	
	Average	0.804	0.155	0.852	0.804	0.803	0.658	0.959	0.955	
Bootstrap Aggregation	Normal	0.917	0.249	0.736	0.917	0.816	0.663	0.928	0.909	0.822
	Anomaly	0.751	0.083	0.923	0.751	0.828	0.663	0.928	0.916	
	Average	0.822	0.155	0.842	0.822	0.823	0.663	0.928	0.913	
Adaptive Boosting	Normal	0.968	0.399	0.648	0.968	0.776	0.589	0.935	0.919	0.822
	Anomaly	0.601	0.032	0.961	0.601	0.74	0.589	0.935	0.941	
	Average	0.759	0.19	0.826	0.759	0.755	0.589	0.935	0.932	
Bayesian Network	Normal	0.973	0.429	0.632	0.973	0.766	0.57	0.945	0.94	0.759
	Anomaly	0.571	0.027	0.965	0.571	0.718	0.57	0.945	0.955	
	Average	0.744	0.2	0.822	0.744	0.739	0.57	0.945	0.949	
Naive Bayes	Normal	0.931	0.367	0.657	0.931	0.771	0.572	0.895	0.844	0.761
	Anomaly	0.633	0.69	0.924	0.633	0.751	0.572	0.914	0.911	
	Average	0.761	0.198	0.809	0.761	0.759	0.572	0.908	0.882	
SVM	Normal	0.954	0.355	0.670	0.954	0.787	0.608	0.799	0.659	0.77
	Anomaly	0.645	0.046	0.948	0.645	0.768	0.608	0.799	0.814	
	Average	0.778	0.179	0.829	0.778	0.776	0.608	0.799	0.747	
Synthetic data	Normal	0.922	0.302	0.698	0.922	0.794	0.620	0.916	0.901	0.794
	Anomaly	0.698	0.078	0.922	0.698	0.794	0.620	0.918	0.913	
	Average	0.794	0.175	0.825	0.794	0.794	0.620	0.917	0.908	

**Table 10:** Results when analyzing NSL-KDD’9$9^{-21}$.

Classifier	Class	TPR	FPR	Precision	Recall	F-Measure	MCC	AUC	PRC Area	Accuracy
Decision Stump	Normal	0.848	0.416	0.311	0.848	0.456	0.33	0.716	0.292	0.631
	Anomaly	0.584	0.152	0.945	0.584	0.722	0.33	0.716	0.893	
	Average	0.632	0.2	0.83	0.632	0.674	0.33	0.716	0.783	
RepTree	Normal	0.635	0.342	0.292	0.963	0.4	0.231	0.751	0.372	0.643
	Anomaly	0.658	0.365	0.89	0.658	0.757	0.231	0.751	0.923	
	Average	0.654	0.361	0.782	0.654	0.692	0.231	0.751	0.823	
Random Forest	Normal	0.875	0.425	0.314	0.875	0.462	0.347	0.794	0.576	0.629
	Anomaly	0.575	0.125	0.954	0.575	0.718	0.347	0.794	0.935	
	Average	0.63	0.179	0.838	0.63	0.671	0.347	0.794	0.87	
Bootstrap Aggregation	Normal	0.637	0.35	0.281	0.637	0.396	0.225	0.743	0.465	0.647
	Anomaly	0.65	0.363	0.89	0.65	0.751	0.225	0.743	0.922	
	Average	0.647	0.361	0.78	0.647	0.687	0.225	0.743	0.839	
Adaptive Boosting	Normal	0.866	0.518	0.217	0.866	0.413	0.272	0.724	0.394	0.522
	Anomaly	0.482	0.134	0.942	0.482	0.638	0.272	0.724	0.901	
	Average	0.552	0.204	0.82	0.552	0.597	0.272	0.724	0.809	
Bayesian Network	Normal	0.878	0.563	0.257	0.878	0.398	0.25	0.744	0.486	0.516
	Anomaly	0.437	0.122	0.942	0.437	0.597	0.25	0.744	0.928	
	Average	0.517	0.202	0.817	0.517	0.561	0.25	0.744	0.848	
Naive Bayes	Normal	0.678	0.469	0.243	0.678	0.358	0.161	0.648	0.294	0.557
	Anomaly	0.531	0.322	0.882	0.531	0.663	0.161	0.65	0.876	
	Average	0.558	0.348	0.766	0.558	0.607	0.161	0.65	0.77	
SVM	Normal	0.180	0.001	0.982	0.180	0.304	0.385	0.589	0.325	0.850
	Anomaly	0.999	0.820	0.846	0.999	0.916	0.385	0.589	0.846	
	Average	0.851	0.672	0.871	0.851	0.805	0.385	0.589	0.752	
Synthetic data	Normal	0.905	0	1	0.095	0.905	N/A	N/A	N/A	0.899
	Anomaly	0	0.095	0	0	0	N/A	N/A	N/A	
	Average	0.905	0	1	0.095	0.95	N/A	N/A	N/A	

**Table 11:** Comparison with related works in terms of accuracy.

Method	NSL-KDD’9$9^{+}$(%)	NSL-KDD’9$9^{-21}$(%)
J48	81.05	63.97
Naive Bayes	76.56	55.77
NB tree	82.02	66.16
Random forests	80.67	63.25
Random tree	81.59	58.51
M-L perceptron	77.41	57.34
SVM	69.52	42.29
Fuzzy	82.41	67.06
Fuzzy D&D	84.12	68.82
**This proposal (Classification)**	**82.2**	**64.7**

**Table 12:** Forecasting results for each horizon.

Forecasting Horizon (H)	Selected Algorithm	Parameter Calibration	SMPAPE
1	Multiplicative Holt-Winters	alpha = 0.5, beta = 0.1, gamma = 0.9	0.0004
5	Multiplicative Holt-Winters	alpha = 0.1, beta = 0.3, gamma = 0.9	0.6972
10	Additive Holt-Winters	alpha = 0.1, beta = 0.3, gamma = 0.1	1.7622
